# Temporal Dynamics and Bidirectional Longitudinal Association Between Physical Function and Depressive Symptoms in Older Adults

**DOI:** 10.1002/jcsm.70223

**Published:** 2026-01-30

**Authors:** Belayneh Mengist, Mojtaba Lotfaliany, Julie A. Pasco, Bruno Agustini, Michael Berk, Suzanne G. Orchard, Joanne Ryan, Alice J. Owen, Robyn L. Woods, John J. McNeil, Mohammadreza Mohebbi

**Affiliations:** ^1^ School of Medicine Deakin University, the Institute for Mental and Physical Health and Clinical Translation (IMPACT) Geelong Victoria Australia; ^2^ College of Medicine and Health Sciences Debre Markos University Debre Markos Ethiopia; ^3^ School of Public Health and Preventive Medicine Monash University Melbourne Victoria Australia; ^4^ Department of Medicine–Western Health The University of Melbourne St Albans Victoria Australia; ^5^ Biostatistics Unit, Faculty of Health Deakin University Geelong Victoria Australia

**Keywords:** depression, mental health, physical function, physical health

## Abstract

**Background:**

Physical function decline and depression are major challenges of the rapidly growing older population. This longitudinal study aimed to investigate the bidirectional longitudinal association between physical function and depressive symptoms, which are poorly understood in older adults.

**Methods:**

We utilised data from the ASPirin in Reducing Events in the Elderly (ASPREE) clinical trial and extended follow‐up cohort between 2010 and 2022. The presence of depressive symptoms was defined as a score of ≥ 8 on the Center for Epidemiologic Studies Depression 10‐item scale. Physical function was assessed using gait speed for physical performance and handgrip strength for muscle strength. Gait speed and handgrip strength were categorised using the European Working Group on Sarcopenia in Older People (EWGSOP2) cut‐off points. The bidirectional association between physical function and depressive symptoms was estimated using generalised estimating equations models. The robustness of the longitudinal bidirectional relationship was assessed using random‐intercept cross‐lagged panel models.

**Results:**

Among 19 114 ASPREE participants, 15 854 (56% females) older adults (mean age 75 years) were included in the analysis. During a median follow‐up of 8.4 years, participants with combined poor physical performance and weak muscle strength had 81% higher odds of developing depressive symptoms compared to those with good performance and strength (OR = 1.81, 95% CI: 1.55–2.11). Conversely, participants with depressive symptoms had 70% higher odds of reduced physical function (combined poor physical performance and weak muscle strength) compared to those with no depressive symptoms (OR = 1.70, 95% CI: 1.61–1.80). The random‐intercept cross‐lagged panel models verified the bidirectional longitudinal associations between physical function and depressive symptoms across the follow‐up waves.

**Conclusions:**

Reduced physical function and depressive symptoms are associated bidirectionally over time of a similar magnitude. Understanding this reciprocal association is crucial for developing effective prevention and treatment strategies for physical and mental health conditions.

## 1 Introduction

Physical function is a critical component of healthy ageing and a key indicator of health status in older adults [1]. Declines in physical function, such as lower gait speed and weak handgrip strength, are diagnostic features of severe sarcopenia and are markers of frailty [2]. As people get older, significant physical changes occur, including muscle weakness, slowness, lower reserve capacity and osteoporosis [3]. The decline in physical function is associated with many adverse health outcomes, such as falls, fractures, hospitalisation, lower health‐related quality of life, reduced activities of daily living and increased risk of death [4]. It is also associated with loss of independence and considerable healthcare costs [5].

Depression in later life is a major public health concern affecting a substantial portion of older adults and is considered the second leading cause of disability [6]. A meta‐analysis indicates that the global prevalence of late‐life depression is 28.4% [7]. Depression in older adults is often under‐recognised, linked to poor treatment adherence and a high rate of recurrence associated with various adverse health outcomes, including cognitive impairment, physical disability and mortality [6].

Consequently, declines in both physical function and depression are major challenges of the rapidly growing older population and may be fundamentally interrelated. Late‐life depression contributes to the ageing process and adversely affects physical health and daily life functions [8], while individuals with poor physical function often experience depression [9]. Prior studies suggest that older adults with declining physical function tend to develop depression [10, 11]. Conversely, people with mental health problems, such as depression, may be more likely to experience functional decline, such as slowness and weakness [9].

Previous studies examining the association between physical function and depression in older adults have often relied on data from a single time point, small sample sizes, inadequate control for confounders and relatively short follow‐up periods [12, 13, 14]. In addition, the temporal order and direction of this association remain uncertain as well as whether the relationship persists over time. The question: *Does a decline in physical function influence the risk of depression or does the reverse association better explain the link?* remains unanswered. Therefore, the current study aimed to investigate the bidirectional associations between physical function and depressive symptoms and to quantify the bidirectional changes over time using a large sample of repeatedly measured longitudinal data among community‐dwelling older adults. In addition, the bidirectional association between physical function and mental health quality of life was investigated. We also aimed to explore potential modifying effects of physical activity and diet quality on the association between physical function and depressive symptoms.

## 2 Methods

### 2.1 Data Source and Study Participants

**FIGURE 1:**
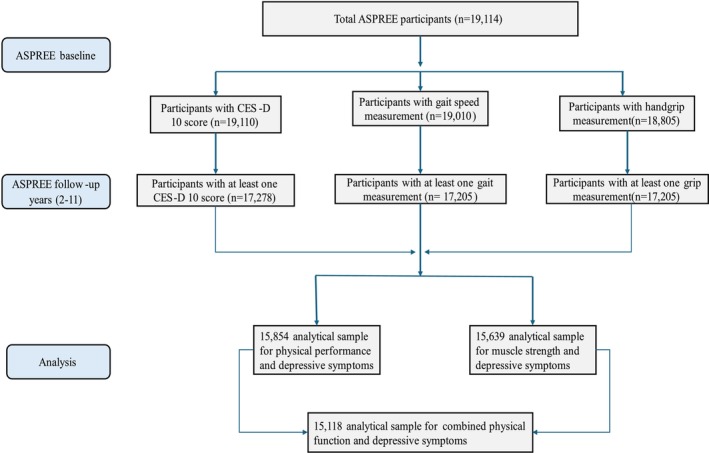
Flowchart showing the selection process of the analytical sample in ASPREE.

We used data from the ASPirin in Reducing Events in the Elderly (ASPREE) extended cohort study. The ASPREE study was a double‐blind, randomised, placebo‐controlled clinical trial of daily 100 mg enteric‐coated aspirin in 19 114 community‐dwelling older adults aged ≥ 70 years from Australia and the United States of America (USA) (≥ 65 years for the US minorities). Participants free from overt cardiovascular disease, dementia, independence‐limiting physical disability and serious illness likely to cause death within 5 years were recruited between 2010 and 2014. The clinical trial was completed in June 2017 and willing participants were followed up after the trial phase through the ASPREE‐eXTension (ASPREE‐XT) observational study until 2024. The details of the ASPREE and ASPREE‐XT studies are found elsewhere [15, 16]. All participants provided written informed consent. The selection process of the analytical sample is presented in the study flowchart (Figure 1). In this analysis, we used data from between 2010 and 2022.

### 2.2 Study Variables

#### 2.2.1 Depressive Symptoms

Depressive symptoms were measured using the Center for Epidemiologic Studies—Depression 10 (CES‐D 10) scale. The CES‐D 10 is a self‐administered questionnaire of 10 items and the total score ranges from 0 to 30, with higher scores indicating more severe depressive symptoms. It is a valid and reliable instrument used to assess clinically relevant depressive symptoms [17]. The presence of clinically relevant depressive symptoms was defined by a total score of ≥ 8 on the CES‐D 10 scale [18].

#### 2.2.2 Mental Health Quality of Life

Mental health quality of life, measured using the 12‐item Short Form Health Survey (SF‐12), has been considered a proxy measure of depression [19]. The SF‐12 Mental Component Score (MCS) was generated by combining the items using the USA general population‐derived item weights and standardised with a mean value of 50 and a standard deviation of 10 [20]. The score ranges from 0 to 100, with higher values indicating better mental health quality of life. Previous research has explored the convergent validity between the CES‐D 10 and the SF‐12 MCS, suggesting that both instruments are correlated [18]. The SF‐12 MCS has been shown to identify depression with a sensitivity of 87% and a specificity of 83% [21].

Both the CES‐D 10 and the SF‐12 were administered annually from the ASPREE baseline visit through the ASPREE‐XT study.

#### 2.2.3 Physical Function

Physical function was assessed via gait speed for physical performance and handgrip strength for muscle strength. Gait speed and handgrip strength were administered at every even‐year and close out ASPREE data collection visits and annually in the ASPREE‐XT study.

For gait speed, the average time in seconds was measured as the time to walk 3 m at a habitual pace indoors on a flat surface from a standing start, with at least 1 m spare at the end to ensure the usual pace was maintained over the full course [22]. The gait speed was calculated as distance (3 m)/time in seconds for analysis. The mean gait speed of the two measures was calculated and dichotomised into good physical performance (> 0.8 m/s) and poor physical performance (≤ 0.8 m/s) according to the European Working Group on Sarcopenia in Older People (EWGSOP2) cut‐off points [2].

Handgrip strength was assessed in kilogram‐force (kgf) using a hydraulic hand dynamometer (Jamar; Lafayette Instruments) and recorded following the American Society of Hand Therapists (ASHT) guidelines [22]. Three tests for each hand (right and left) were taken and the average value for each hand was calculated. The strongest (dominant hand) grip measurement was used for analysis.

Handgrip strength was categorised into weak muscle strength (< 27 kg for males and < 16 kg for females) and good muscle strength using the cut‐off points described in the EWGSOP2 [2].

Overall, physical function was assessed by combining the two physical measures (physical performance and muscle strength). Participants were classified as: (i) both poor performance and weak strength, (ii) poor performance and good strength, (iii) good performance and weak strength, (iv) good performance and good strength.

Poor physical function was examined both as a risk factor and an outcome of depressive symptoms for up to 11 years of follow‐up.

### 2.3 Covariates

Socio‐demographic factors (age, sex, racial category (white vs. non‐white), years of education (≤ 12 years and > 12 years) and living arrangement (at home alone vs. living with family or friends)) were collected at the study baseline. Smoking status and alcohol use were categorised into current, former and never. Body mass index (BMI) in kg/m^2^, baseline global cognitive function (Modified Mini‐Mental State examination; 3MS), multimorbidity (coexistence of two or more chronic conditions such as hypertension, diabetes mellitus, dyslipidaemia, chronic kidney disease (CKD), gastro‐oesophageal reflux disease (GORD), pulmonary disease, gout and Parkinson's disease) and polypharmacy (use of five or more prescription medications) were included. In addition, variables such as social support, diet quality, intensity of physical activity and level of pain were used from an ASPREE sub‐study; ASPREE Longitudinal Study of Older Persons (ALSOP) of Australian participants [23]. The dietary inflammatory score [24] was used as a proxy for diet quality (i.e., anti‐inflammatory versus pro‐inflammatory). A directed acyclic graph (DAG) conceptualising the relationship of covariates with physical function and depressive symptoms is presented in Figure S1.

### 2.4 Statistical Analysis

Baseline characteristics of participants were summarised by frequencies and percentages for categorical variables and means with standard deviations for continuous variables. The rates of depression and physical function decline (decline in gait speed and grip strength) over follow‐up years were presented graphically. Generalised estimating equations (GEE) models were fitted to examine the longitudinal bidirectional relationship between physical function and depression using a logit link function and a first‐order autoregressive correlation structure to account for autocorrelation in repeated measures.

To investigate depressive symptoms in association with physical function, depressive symptoms (i.e., CES‐D 10 ≥ 8) measured at each annual visit from year two onwards were used as a binary outcome. Physical function (including gait speed, grip strength and their combination) was treated as a time‐varying exposure from baseline and throughout annual visits. To examine the association between physical function and depressive symptoms, follow‐up measures of gait speed and grip strength from year two onwards were used as binary outcomes. The presence of depressive symptoms was treated as a time‐varying exposure from baseline and across annual visits. The year‐one visit was excluded because no physical function measurements were available. We estimated the change in time effects on model‐adjusted associations by including two‐way interactions between nominal time (i.e., annual follow‐up) with time‐varying exposures. To investigate the bidirectional association between physical function and mental component quality of life, GEE models were applied using repeated measures of categorical physical function and continuous MCS. The Quasi‐likelihood under the Independence model Criterion (QIC) was used to select the best‐fitting model and working correlation structure, with the model yielding the smallest QIC value preferred.

Ordinal logistic regression with a Huber robust estimator to correct the model standard error, considering clustering for repeated measures, was used to estimate the odds of physical function categories (good functioning, either poor physical performance or weak muscle strength, and both poor performance and weak muscle strength) as an ordinal outcome using depressive symptom and MCS as exposure variables. A proportional odds ratio is a statistical measure derived from ordinal logistic regression. It assesses how the odds of an outcome being at or below a specified threshold change relative to being above that threshold for a one‐unit increase in a predictor variable, assuming the relationship remains constant across all levels of the outcome variable.

Potential effect modification of diet quality (based on dietary inflammatory score), usual physical activity intensity and level of pain [25], on the association between physical function and depression was investigated using data from the ALSOP sub‐study (Australian participants). Sensitivity analyses were conducted by excluding participants with depressive symptoms and poor physical function at baseline. Subgroup analyses were also performed based on sex category. Odds ratios (OR) and 95% confidence intervals (CI) were reported.

To evaluate the dynamic bidirectional associations between physical function and depressive symptoms over the follow‐up time, a random‐intercept cross‐lagged panel model (RI‐CLPM) was applied using the *lavaan* package in R. In the RI‐CLPM, autoregressive paths reflect the stability of each variable across time points and the cross‐lagged paths estimate the directional influence of one variable on another. The model thus enables simultaneous testing of both the effect of each variable on itself over time and the cross‐time influence on the other variable [26]. Biennial waves (baseline, Year 2, Year 4 and Year 6) were included in the cross‐lagged panel modelling to optimise the use of the available sample. The full information maximum‐likelihood method was used and standardised path coefficients (*B*) were reported. Overall model fit was assessed using the Comparative Fit Index (CFI), Tucker–Lewis Index (TLI), root mean square error of approximation (RMSEA) and standardised root mean square residual (SRMR) [27]. A two‐tailed *p*‐value of less than 0.05 was considered statistically significant. Stata (Version 18, StataCorp, College Station, Texas, USA) and R version 4.4.2 (R Project for Statistical Computing) were used for analyses.

## 3 Results

Of the total 19 114 participants enrolled in the ASPREE study, 15 854 were included in the final analysis. The follow‐up period had a median (IQR) of 8.4 (2.4) years. The analytical samples for each outcome variable are presented in the study flowchart (Figure 1). The mean age of participants at baseline was 75 years (range: 65–98 years) and 56% of the participants were females. Female participants were more likely to have lower physical function and higher depressive symptoms, while younger participants tended to have better physical function. At baseline, participants with poor physical function and depressive symptoms had higher rates of multimorbidity and polypharmacy. The baseline characteristics of participants are shown in Table 1.

**TABLE 1:** Baseline characteristics of the study participants.

	Poor physical performance		Weak muscle strength		Depressive symptoms (CES‐D 10 ≥ 8)	
	Yes (3026)	No (15984)	Yes (3224)	No (15581)	Yes (1879)	No (17231)
Age: mean (SD)	77.0 (5.8)	74.8 (4.2)	77.1 (5.8)	74.7 (4.2)	75.2 (4.7)	75.1 (4.5)
Sex: female, *n* (%)	2148 (71.0)	8570 (53.6)	1917 (59.5)	8666 (55.6)	1248 (66.4)	9531 (55.3)
Education status: ≤ 12 years	1880 (62.1)	9012 (56.4)	1878 (58.3)	8884 (57.0)	1151 (61.3)	9800 (56.9)
Alcohol use, *n* (%)						
Current	1958 (64.7)	12 604 (78.9)	2349 (72.9)	12 066 (77.4)	1365 (72.6)	13 273 (77.0)
Former	278 (9.2)	855 (5.3)	211 (6.5)	902 (5.8)	155 (8.3)	981 (5.7)
Never	790 (26.1)	2524 (15.8)	663 (20.6)	2613 (16.8)	359 (19.1)	2977 (17.3)
Smoking, *n* (%)						
Current	175 (5.8)	551 (3.5)	119 (3.7)	603 (3.9)	110 (5.9)	624 (3.6)
Former	1141 (37.7)	6612 (41.4)	1239 (38.4)	6437 (41.3)	756 (40.2)	7041 (40.9)
Never	1710 (56.5)	8817 (55.1)	1865 (57.9)	8541 (54.8)	1013 (53.9)	9566 (55.5)
BMI (kg/m^2^): mean (SD)	29.6 (5.9)	27.8 (4.4)	27.9 (5.0)	28.1 (4.7)	28.7 (5.4)	28.0 (4.6)
Baseline cognition (3MS): mean (SD)	92.1 (5.1)	93.7 (4.5)	92.8 (5.0)	93.6 (4.5)	92.9 (4.7)	93.5 (4.6)
Living arrangement: living at home with family or friends	1695 (56.0)	11 019 (68.9)	1937 (60.1)	10 660 (68.4)	1063 (56.6)	11 714 (68.0)
Racial category: white	2451 (82.5)	15 151 (95.6)	2946 (92.7)	14 486 (93.8)	1687 (90.6)	16 007 (93.9)
Multimorbidity: yes	2422 (80.0)	11 852 (74.1)	2490 (77.2)	11 628 (74.6)	1520 (80.9)	12 831 (74.5)
Polypharmacy: yes	943 (31.2)	2874 (18.0)	846 (26.2)	2921 (18.7)	553 (29.4)	3288 (19.1)

*Note:* Poor physical performance: gait speed ≤ 0.8 m/s. Weak muscle strength: handgrip strength < 27 kg for males and < 16 kg for females. Multimorbidity: coexistence of two or more chronic health conditions (hypertension, diabetes mellitus, dyslipidaemia, chronic kidney disease, gastro‐oesophageal reflux disease, respiratory condition, osteoarthritis, gout and Parkinson's disease). Polypharmacy: taking ≥ 5 prescription medications daily.

The proportion of dropouts during the follow‐up (Waves 2–11) after baseline was 9.6% for depressive symptoms and 10.2% for physical function (gait and grip combined). A detailed breakdown of missingness by baseline variables is provided in Supplementary Table 1. In summary, compared to the complete group, the dropout group was older at baseline and had a higher prevalence of current smokers and morbidities. The complete group had a higher baseline 3MS score.

### 3.1 Change in Physical Function and Depressive Symptoms Over Time

**FIGURE 2:**
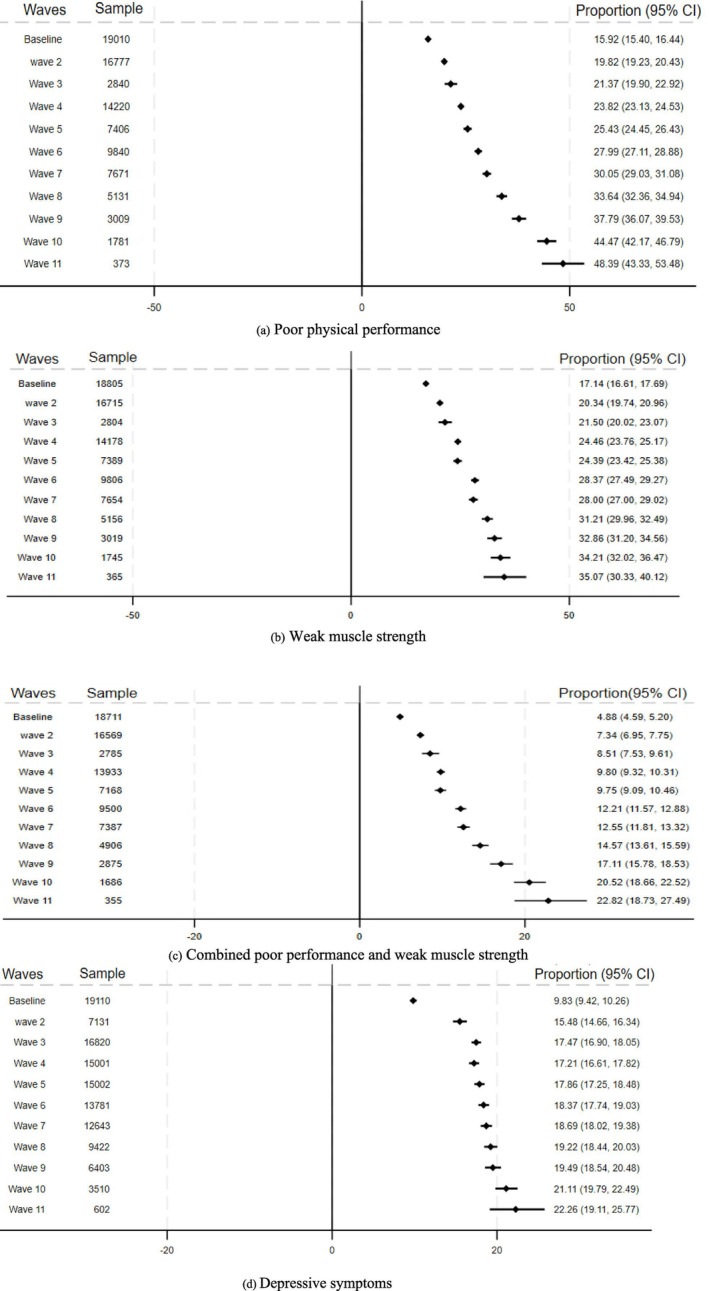
Proportion of study population with (a) poor physical performance (b) weak muscle strength (c) combined poor physical performance and weak muscle strength (d) depressive symptoms.

At baseline, 15.9%, 17.1% and 9.8% of the participants had poor physical performance, weak muscle strength and depressive symptoms, respectively. These proportions increased progressively over time and by year seven the prevalence of poor physical performance, weak muscle strength and depressive symptoms reached 30.0%, 28.0% and 18.6%, respectively. By the end of the follow‐up of the current study (Year 11), the prevalence of poor physical performance, weak muscle strength and depressive symptoms had increased to 48.3%, 35.0% and 22.2%, respectively (Figure 2).

### 3.2 Associations of Physical Function Exposure and Depressive Symptoms Outcome in Older Adults

Table 2 presents the association between physical function and depressive symptoms during the follow‐up period. In the fully adjusted GEE models, participants with poor physical performance exhibited 57% higher odds of depressive symptoms compared to those with good physical performance (OR = 1.57, 95% CI: 1.41–1.74). Similarly, participants with weak muscle strength had 26% higher odds of depressive symptoms compared to those with good muscle strength (OR = 1.26, 95% CI: 1.13–1.40). Combining the two physical function measures strengthened their association with the presence of depressive symptoms: those with combined poor physical performance and weak muscle strength had 81% higher odds of depressive symptoms compared to those with both good physical performance and muscle strength (OR = 1.81, 95% CI: 1.55–2.11).

**TABLE 2:** Association between physical performance, muscle strength and depressive symptoms estimated from adjusted GEE models of repeated measure exposure and outcome.

Physical function		Depressive symptoms		Mental component score	
		OR (95% CI)	*p*	Mean difference (95% CI)	*p*
Physical performance	Poor	1.57 (1.41–1.74)	< 0.001	−1.21 (−1.65 to −0.78)	< 0.001
	Good	Reference		Reference	
Muscle strength	Weak	1.26 (1.13–1.40)	< 0.001	−0.82 (−1.25 to −0.39)	< 0.001
	Good	Reference		Reference	
Combination of physical performance and muscle strength	Both weak and poor	1.81 (1.55–2.11)	< 0.0001	−1.76 (−2.39 to −1.12)	< 0.0001
	Poor performance only	1.50 (1.32–1.70)		−1.09 (−1.60 to −0.58)	
	Weak strength only	1.18 (1.02–1.36)		−0.44 (−0.92 to 0.04)	
	Neither weak nor poor (both good physical function)	Reference (1)		Reference (0)	

*Note:* Poor physical performance: gait speed ≤ 0.8 m/s. Weak muscle strength: handgrip strength < 27 kg for males and < 16 kg for females. Models were adjusted for age, sex, educational status, alcohol use, BMI, smoking, baseline cognition, living arrangement, racial category, multimorbidity and polypharmacy. The interaction between exposure and wave was not significant.

The mean difference for the SF‐12 MCS was significantly decreased among participants with poor physical performance (*β* = −1.21; 95% CI: −1.65 to −0.78) and weak muscle strength (*β* = −0.82; 95% CI: −1.25 to −0.39).

### 3.3 Associations of Depressive Symptoms Exposure and Physical Function Outcome in Older Adults

As shown in Table 3, the presence of depressive symptoms determined the longitudinal status of physical function. In the fully adjusted GEE models, participants with depressive symptoms had 47% higher odds of poor physical performance compared to those without depressive symptoms (OR = 1.47, 95% CI: 1.33–1.62). Similarly, participants with depressive symptoms had 11% higher odds of weak muscle strength compared to those with no depressive symptoms (OR = 1.11, 95% CI: 1.02–1.21).

**TABLE 3:** Association between depressive symptoms and physical function as a repeated measure exposure and outcome.

Depressive symptoms (CES‐D 10 ≥ 8)		Poor physical performance		Weak muscle strength		Decline in physical function^a^	
		OR (95% CI)	*p*	OR (95% CI)	*p*	POR (95% CI)	*p*
Depressive symptoms	Yes	1.47 (1.33–1.62)	< 0.001	1.11 (1.02–1.21)	0.017	1.70 (1.61–1.80)	< 0.001
	No	Reference (1)		Reference (1)		Reference (1)	
Mental component score	0.98 (0.97–0.987)		< 0.001	0.989 (0.98–0.99)	0.001	0.98 (0.977–0.983)	< 0.001

*Note:* Poor physical performance: gait speed ≤ 0.8 m/s. Weak muscle strength: handgrip strength < 27 kg for males and < 16 kg for females. Models were adjusted for age, sex, educational status, alcohol use, BMI, smoking, baseline cognition, living arrangement, racial category, multimorbidity and polypharmacy.

Abbreviation: POR: proportional odds ratio.

^a^ Decline in physical function as ordinal outcome (good/normal physical function, poor physical performance or weak muscle strength and both poor performance and weak strength).

In the fully adjusted ordinal logistic regression model, the odds of physical function decline increased by 70% among participants with depressive symptoms, moving from one level of physical function to a more severe level proportionally (POR = 1.70, 95% CI: 1.61–1.80).

The two‐way interaction between exposure variables and follow‐up wave was not significant. Sub‐group analyses using sex categories showed consistent findings with the main analyses (Tables S2a and S2b). In addition, sensitivity analyses excluding participants with depressive symptoms and poor physical function at baseline showed consistent findings (Tables S3 and S4). Sensitivity analyses adjusting for social support for Australian participants showed consistent findings (Table S5).

In the interaction analysis, we found no significant interaction effects of diet quality, physical activity intensity or pain levels on the bidirectional association between physical function and depressive symptoms, indicating that the observed bidirectional associations were not modified by diet quality, intensity of physical activity or levels of pain (Table S6).

### 3.4 RI‐CLPM for Physical Performance and Depressive Symptoms

**FIGURE 3:**
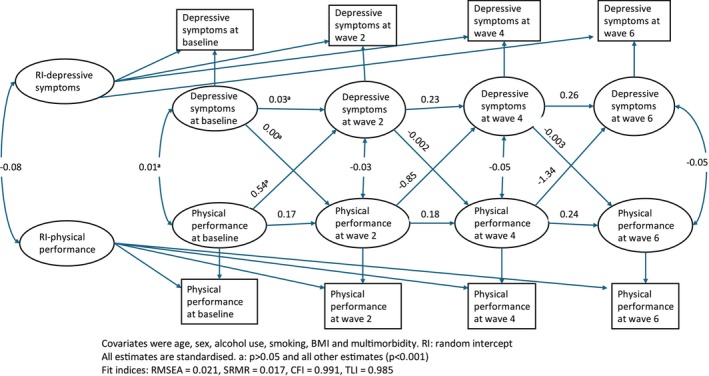
Random intercept cross‐lagged panel model (RI‐CLPM) of the bidirectional longitudinal association between physical performance and depressive symptoms.

Figure 3 shows the standardised path coefficients of the RI‐CLPM, showing a bidirectional association between physical performance and depressive symptoms.

The RI‐CLPM illustrated that physical performance and depressive symptoms were inversely associated in the subsequent waves; physical performance at Wave 2 and depressive symptoms at Wave 4 (*B* = −0.85; 95% CI −1.41 to −0.28), physical performance at Wave 4 and depressive symptoms at Wave 6 (*B* = −1.34; 95% CI, −1.92 to −0.75), depressive symptoms at Wave 2 and physical performance at Wave 4 (*B* = −0.002; 95% CI, −0.004 to −0.001) and depressive symptoms at Wave 4 and physical performance at Wave 6 (*B* = −0.003; 95% CI, −0.005 to −0.002). However, the cross‐lagged path estimates indicated no association between physical performance at baseline and depressive symptoms at Wave 2 (*B* = 0.54; 95% CI −0.17 to 1.25) and depressive symptoms at baseline and physical performance at Wave 2 (*B* = 0.00; 95% CI −0.002 to 0.002).

The auto‐regressive paths for physical performance indicated that prior physical performance was significantly associated with subsequent physical performance. Similarly, the auto‐regressive paths for depressive symptoms were significant, except for baseline to Wave 2, indicating that depressive symptoms at one time point predicted their subsequent values. The covariance between the latent random intercepts showed the between‐person associations between depressive symptoms and physical performance (*B* = −0.082; 95% CI −0.091 to −0.073) (Figure 3 and Table 4).

**TABLE 4:** Random intercept cross‐lagged panel model (RI‐CLPM) longitudinal association between physical function (physical performance and muscle strength) and depressive symptoms.

Characteristic	Standardised coefficient (95% CI)	*p*		Standardised coefficient (95% CI)	*p*
Between‐person covariance					
RI‐physicalP **↔** RI‐ depressiveS	−0.082 (−0.091 to −0.073)	< 0.0001	RI‐MuscleS **↔** RI‐depressiveS	−1.660 (−1.950 to −1.370)	< 0.001
Within‐person covariance					
PhysicalP‐baseline ↔ depressiveS‐baseline	0.009 (0.00 to 0.018)	0.052	MuscleS baseline **↔** depressiveS baseline	0.492 (0.240 to 0.743)	< 0.001
PhysicalP w2 **↔** depressiveS w2	−0.030 (−0.044 to −0.015)	< 0.0001	MuscleS w2 **↔** depressiveS w2	−0.386 (−0.815 to 0.042)	0.077
PhysicalP w4 **↔** depressiveS w4	−0.048 (−0.058 to −0.038)	< 0.0001	MuscleS w4 **↔** depressiveS w4	−0.807 (−1.075 to −0.539)	< 0.001
PhysicalP w6 **↔** depressiveS w6	−0.050 (−0.060 to −0.040)	< 0.0001	MuscleS w6 **↔** depressiveS w6	−0.593 (−0.858 to −0.327)	< 0.001
Within‐person regression paths					
PhysicalP baseline → PhysicalP w2	0.175 (0.151 to 0.199)	< 0.0001	MuscleS baseline → MuscleS w2	0.129 (0.102 to 0.155)	< 0.001
PhysicalP W2 → PhysicalP w4	0.186 (0.157 to 0.215)	< 0.0001	MuscleS W2 → MuscleS w4	0.188 (0.149 to 0.228)	< 0.001
PhysicalP W4 → PhysicalP w6	0.239 (0.208 to 0.270)	< 0.0001	MuscleS W4 → MuscleS w6	0.311 (0.275 to 0.347)	< 0.001
DepressiveS baseline → depressiveS w2	0.029 (−0.030 to 0.088)	0.331	DepressiveS baseline → depressiveS w2	0.034 (−0.024 to 0.093)	0.254
DepressiveS w2 → depressiveS w4	0.234 (0.201 to 0.267)	< 0.0001	DepressiveS w2 → depressiveS w4	0.237 (0.204 to 0.270)	< 0.001
DepressiveS w4 → depressiveS w6	0.263 (0.241 to 0.285)	< 0.0001	DepressiveS w4 → depressiveS w6	0.266 (0.244 to 0.288)	< 0.001
PhysicalP baseline → depressiveS w2	0.541 (−0.169 to 1.250)	0.135	MuscleS baseline → depressiveS w2	0.003 (−0.029 to 0.034)	0.870
DepressiveS baseline → PhysicalP w2	−0.000 (−0.002 to 0.002)	0.796	DepressiveS baseline → MuscleS w2	−0.016 (−0.062 to 0.029)	0.485
PhysicalP w2 → depressiveS w4	−0.847 (−1.413 to −0.281)	< 0.001	MuscleS w2 → depressiveS w4	−0.061 (−0.089 to −0.032)	< 0.001
DepressiveS w2 → PhysicalP w4	−0.002 (−0.004 to −0.001)	0.009	DepressiveS w2 → muscleS w4	−0.023 (−0.073 to 0.026)	0.356
PhysicalP w4 → depressiveS w6	−1.339 (−1.924 to −0.755)	< 0.001	MuscleS w4 → depressiveS w6	−0.041 (−0.066 to −0.016)	0.002
DepressiveS w4 → PhysicalP w6	−0.003 (−0.005 to −0.002)	< 0.001	DepressiveS w4 → muscleS w6	−0.042 (−0.074 to −0.010)	0.010

*Note:* ↔ indicates within‐wave covariance between random intercepts or residuals. → indicates lagged regression path estimates. All paths are adjusted for stability between‐person differences via random intercepts.

Abbreviations: DepressiveS: depressive symptoms, physicalP: physical performance, muscleS: muscle strength, RI: random intercept, w: wave.

### 3.5 RI‐CLPM for Depressive Symptoms and Muscle Strength

**FIGURE 4:**
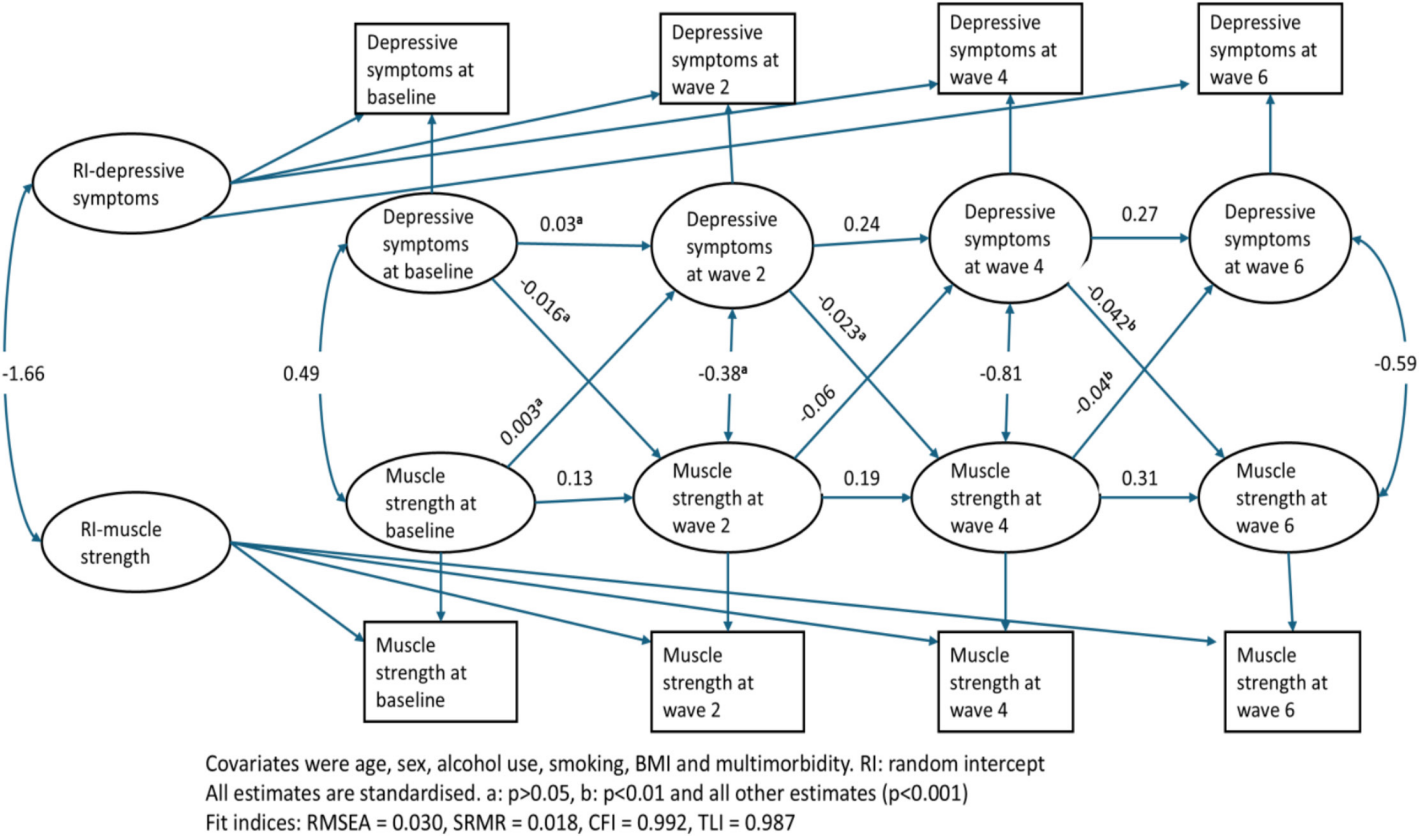
Random intercept cross‐lagged panel model (RI‐CLPM) of the bidirectional longitudinal association between muscle strength and depressive symptoms.

As shown in Figure 4, the RI‐CLPM shows the association between muscle strength and depressive symptoms across the four biennial waves. Muscle strength at Wave 2 was inversely associated with depressive symptoms at Wave 4 (*B* = −0.06; 95% CI −0.09 to −0.03). Similarly, muscle strength at Wave 4 was inversely associated with depressive symptoms at Wave 6 (*B* = −0.04; 95% CI −0.07 to −0.02). On the other hand, depressive symptoms at Wave 4 were inversely associated with muscle strength at Wave 6 (*B* = −0.04; 95% CI, −0.07 to −0.01) and the cross‐lagged path estimates showed no association in the preceding waves.

Muscle strength showed strong temporal continuity as evidenced by the autoregressive effects, ranging from (*B* = 0.13; 95% CI 0.10 to 0.15) to (*B* = 0.31; 95% CI 0.27 to 0.38).

There were also inverse between‐person associations between depressive symptoms and muscle strength as shown by the covariance of the latent random intercepts (*B* = −1.66; 95% CI −1.95 to −1.37) (Table 4).

## 4 Discussion

This study examined the longitudinal bidirectional associations between physical function and depressive symptoms using a large cohort of older adults (age ≥ 65) over 11 years. The results showed that a decline in physical function was associated with developing depressive symptoms. Conversely, depressive symptoms were associated with physical functional decline. The findings are consistent for population average models, i.e., GEEs and dynamic RI‐CLPM models. We found bidirectional relationships between physical function and depressive symptoms in both functional measures (gait speed and grip strength). The rate of both depressive symptoms and physical function decline expectedly increased over the follow‐up period, as biological ageing leads to functional decline. The two measures of physical function (physical performance and muscle strength) were independently associated with depressive symptoms and the association was stronger when the two physical function measures were combined. In the revised European consensus on the definition and diagnosis of sarcopenia, poor physical performance is an indicator of severe sarcopenia and predicts adverse outcomes as a measure of whole‐body function [2]. In our study, the association between poor physical performance and depressive symptoms was more pronounced than the association between weak muscle strength and depressive symptoms.

Previous studies examining the relationship between physical function and depressive symptoms have often relied on cross‐sectional data and primarily focused on unidirectional associations, providing limited evidence for a bidirectional relationship [13, 28, 29, 30]. In our previous study, we reported that both physical performance and muscle strength at baseline predict an increased risk of depression during follow‐up, either independently or in combination [25]. A study from China showed that participants with depressive symptoms were at a higher risk of subsequently developing sarcopenia [28]. Findings from the English Longitudinal Study of Ageing found that individuals with elevated or persistent depressive symptoms had a higher risk of weak grip strength compared to those without depression [29]. Another study using data from the English Longitudinal Study of Ageing also supports a bidirectional inverse relationship between gait speed and depressive symptoms, indicating that slower gait is associated with increased depressive symptoms and vice versa [12]. Consistent with our findings, a Mendelian randomisation study using data from the UK Biobank cohort (aged 48–73 years) reported that weak grip strength and slower usual walking pace had a direct causal effect on depression. However, the reverse association was not significant, suggesting that depression did not causally influence grip strength or walking speed. This discrepancy may be attributed to differences in the study populations, as the UK Biobank included a broader adult age range, whereas the association may be more pronounced in older adults, as in our study [31].

The relationship between physical health and mental health appears fundamentally interdependent, especially in the older population. Physical function decline and depression share common pathophysiological mechanisms, including chronic inflammation, oxidative stress and cellular senescence [32, 33, 34, 35, 36]. These shared pathophysiological features support the bidirectional relationship wherein physical function decline may exacerbate depressive symptoms and, conversely, depression may accelerate functional impairment.

Biologically, elevated levels of pro‐inflammatory cytokines, such as C‐reactive protein (CRP), interleukin‐6 (IL‐6) and tumour necrosis factor‐alpha (TNF‐α), are frequently observed in individuals experiencing psychological stress [28, 31]. These inflammatory markers are associated with depressive symptomatology and impairments in executive function, muscle atrophy and motor speed [28, 32].

Ageing is associated with chronic low‐grade inflammation, musculoskeletal changes and chronic medical conditions that increase depression risk and functional decline [32]. Age‐related physiological changes intrinsic to the ageing process, such as decreased skeletal muscle mass, lead to functional dependency [37, 38]. Ageing is also associated with a decline in brain‐derived neurotrophic factor (BDNF) levels [39]. Lower BDNF levels in older adults may contribute to both depression and physical decline through impaired brain‐muscle communication and reduced capacity for neural repair and adaptation [31, 32].

The bidirectional nature of the association between physical function decline and depressive symptoms can be further explained that poor physical function accelerates biological ageing and accelerated ageing, in turn, increases risk for depressive disorders. Individuals with depressive symptoms are less likely to engage in physical activity and reduced physical activity is a risk factor for functional impairment [28, 40]. Moreover, social determinants such as loneliness, social isolation and reduced social engagement are prevalent in older populations and are established risk factors for both depressive symptoms and functional decline [41]. In our study, the associations remained significant after adjusting for social support.

The associations between physical function and depressive symptoms for baseline‐to‐Wave 2 cross‐lagged paths were nonsignificant, while the GEE models showed that the average across all waves correlations after incorporating a first‐order autoregressive correlation structure were significant. As visualised in the results, a dose–response pattern between depressive symptoms and the proportions of poor physical performance, weak muscle strength and combined poor performance and weak muscle strength was evident across all follow‐up waves. Random fluctuations in the data may contribute to nonsignificant findings at Wave 2; also, the lack of physical functioning measures at Wave 1 may confound the estimated effects at Wave 2. Additionally, the nonsignificant estimates may reflect a healthy cohort effect among participants at baseline, which could translate into a small effect size in the early waves and, as such, low statistical power to detect the impact.

To our knowledge, this study is the first to report a longitudinal bidirectional association between physical function and depressive symptoms in older adults. We applied different statistical approaches that utilise various model assumptions to assess the robustness of the estimates. In addition, the use of objectively measured physical function reduces measurement bias and enhances the validity of the findings. However, the CES‐D 10 is a self‐reported screening tool used to identify clinically relevant depressive symptoms, rather than to provide a clinical diagnosis of depression. In addition, the participants were relatively healthy older adults at baseline and were willing to enrol in a clinical trial with multiple waves of in‐person follow‐up, which may limit the generalisability of the findings to other age groups and to those with more severe chronic health conditions. Although we accounted for a broad range of potential confounders, residual confounding bias cannot be ruled out. Additionally, a model with time‐updated measures of confounders may provide better adjustment for residual confounding. Lastly, loss to follow‐up is common in ageing cohorts and may affect estimates if participants who drop out differ systematically.

## 5 Conclusions

This study reports a bidirectional longitudinal association of similar magnitude between physical function and depressive symptoms in older populations. Our findings suggest that declines in physical function may contribute to the onset or worsening of depressive symptoms and the presence of depressive symptoms may accelerate physical function decline over time. These findings underscore the need for integrated interventions targeting both physical and mental health to promote healthy ageing and prevent disability in later life. Future preventive interventional research targeting the onset of depression or enhancing physical function in older populations can determine causality.

## Funding

The ASPREE clinical trial was supported by the National Institute on Aging and the National Cancer Institute at the National Institutes of Health (U01AG029824 and U19AG062682), the National Health and Medical Research Council (NHMRC) of Australia (334047 and 1127060), Monash University (Australia) and the Victorian Cancer Agency (Australia).

## Ethics Statement

The ASPREE trial was conducted in accordance with the 2008 Declaration of Helsinki and approved by the ethics review board at each participating institution. Monash University Human Research Ethics Committee and the Alfred Hospital Human Research Ethics Committee approved the ALSOP and ASPREE‐XT (Monash 4HREC CF11/1935/2011001094 and Alfred HREC 593/17). All participants provided their written informed consent to participation. The current secondary analysis was approved by the Monash University Human Research Ethics Committee.

## Conflicts of Interest

The authors declare no conflicts of interest.

## Supporting information


**Table S1:** Baseline characteristics of participants, comparing complete cases (at least one follow‐up assessment after baseline) and missing values (those lost to follow‐up).
**Table S2a:** Subgroup analysis by sex showing the association between physical performance and muscle strength and depressive symptoms.
**Table S2b:** Subgroup analysis by sex showing the association between depressive symptoms and physical function.
**Table S3:** Sensitivity analysis showing the association between physical performance, muscle strength and depressive symptoms estimated from adjusted GEE model of repeated measure exposure and outcome after excluding participants with depressive symptoms at baseline.
**Table S4:** Sensitivity analysis showing the association between depressive symptoms and physical function as a repeated measure exposure and outcome after excluding participants with poor physical function at baseline.
**Table S5:** Sensitivity analysis estimating the association between depressive symptoms and physical function, after additionally adjusting for social support (*n* = 10 110).
**Table S6:** Sensitivity analysis showing the association between depressive symptoms and physical function and interaction with diet quality, physical activity and pain symptoms.
**Figure S1:** Factors affecting physical function and depressive symptoms in later life.
**Figure S2:** Proportion of depressive symptoms, slow gait speed and weak grip strength over 11 years of follow‐up.

## Data Availability

Researchers interested in accessing the dataset should request to Monash University at https://aspree.org/aus/for‐researchers/.
