# Supplementary material for: Temporal Dynamics and Bidirectional Longitudinal Association Between Physical Function and Depressive Symptoms in Older Adults
Source: J Cachexia Sarcopenia Muscle. 2026 Jan 30;17(1):e70223. doi: 10.1002/jcsm.70223 (PMC12858256; doi:10.1002/jcsm.70223)
Supplement: Supplementary file 1 — Table S1: Baseline characteristics of participants, comparing complete cases (at least one follow‐up assessment after baseline) and missing values (those lost to follow‐up). Table S2a: Subgroup analysis by sex showing the association between physical performance and muscle strength and depressive symptoms. Table S2b: Subgroup analysis by sex showing the association between depressive symptoms and physical function. Table S3: Sensitivity analysis showing the association between physical performance, muscle strength and depressive symptoms estimated from adjusted GEE model of repeated measure exposure and outcome after excluding participants with depressive symptoms at baseline. Table S4: Sensitivity analysis showing the association between depressive symptoms and physical function as a repeated measure exposure and outcome after excluding participants with poor physical function at baseline. Table S5: Sensitivity analysis estimating the association between depressive symptoms and physical function, after additionally adjusting for social support (n = 10 110). Table S6: Sensitivity analysis showing the association between depressive symptoms and physical function and interaction with diet quality, physical activity and pain symptoms. Figure S1: Factors affecting physical function and depressive symptoms in later life. Figure S2: Proportion of depressive symptoms, slow gait speed and weak grip strength over 11 years of follow‐up. [file JCSM-17-e70223-s001.docx]

**S. Table 1:** Baseline characteristics of participants, comparing complete cases (at least one follow-up assessment after baseline) and missing values (those lost to follow-up)

| **Characteristics** | | **For depressive symptoms outcome** | | | **For physical function (both gait and grip) outcome** | | |
| --- | --- | --- | --- | --- | --- | --- | --- |
|  |  | Complete case | Drop-out | SMD | Complete case | Drop-out | SMD |
| Age: mean (SD) | | 75.0±4.4 | 76.2±5.4 | 0.24 | 75.0±4.4 | 76.0±5.4 | 0.20 |
| Sex: female, n (%) | | 9738 (56.4) | 1044 (56.9) | 0.01 | 9613 (56.0) | 1169 (59.7) | 0.07 |
| Education status: ≤12years, n (%) | | 9776 (56.6) | 1179 (64.2) | 0.15 | 9734 (56.7) | 1221 (62.4) | 0.11 |
| Alcohol use | Current, n (%) | 13358 (77.3) | 1284 (69.9) | 0.13 | 13285 (77.4) | 1357 (69.3) | 0.15 |
|  | Former, n (%) | 955 (5.5) | 181 (9.8) |  | 949 (5.5) | 187 (9.5) |  |
|  | Never, n (%) | 2965 (17.2) | 371 (20.2) |  | 2923 (17.0) | 413 (21.1) |  |
| Smoking | Current, n (%) | 591 (3.4) | 144 (7.8) | 0.18 | 579 (3.4) | 156 (8.0) | 0.16 |
|  | Former, n (%) | 7011 (40.6) | 788 (42.9) |  | 6984 (40.7) | 815 (41.6) |  |
|  | Never, n (%) | 9676 (56.0) | 904 (49.2) |  | 9594 (55.9) | 986 (50.4) |  |
| BMI (kg/m2): mean (SD) | | 28.1±4.7 | 27.9±5.1 | 0.03 | 28.1±4.7 | 28.0±5.1 | 0.01 |
| Baseline cognition (3MS): mean (SD) | | 93.6±4.5 | 91.5±5.2 | 0.42 | 93.6±4.5 | 91.7±5.2 | 0.38 |
| Living arrangement | living at home with family or friends, n (%) | 11659 (67.5) | 1121(61.1) | 0.13 | 11604 (67.6) | 1176 (60.1) | 0.15 |
| Racial category | White, n (%) | 16096 (94.1) | 1602 (88.8) | 0.18 | 16006 (94.2) | 1692 (88.0) | 0.21 |
| Multimorbidity | Yes, n (%) | 12917 (74.8) | 1437 (78.3) | 0.08 | 12821 (74.7) | 1533 (78.3) | 0.08 |
| Polypharmacy | Yes, n (%) | 3535 (20.5) | 307 (16.7) | 0.09 | 3495 (20.4) | 347 (17.7) | 0.06 |

- SMD: standardised mean difference
- We considered complete cases if participants contributed outcome data to at least one follow-up after baseline (from wave 2 to wave 11), included in the analysis (for GEE models), and those with no follow-up data were considered lost to follow-up (drop-out).
- Overall missing: depressive symptoms = 9.6% and physical function = 10.2%

**Subgroup analysis by sex**

**S. Table 2a:** subgroup analysis by sex showing the association between physical performance and muscle strength, and depressive symptoms

| Physical function | | Depressive symptoms | | | | Mental component score | | | |
| --- | --- | --- | --- | --- | --- | --- | --- | --- | --- |
|  |  | Male | | Female | | Male | | Female | |
|  |  | OR (95% CI) | p-value | OR (95% CI) | p-value | Mean difference (95% CI) | p-value | Mean difference (95% CI) | p-value |
| Physical performance | Poor | 1.61 (1.35-1.91) | <0.001 | 1.55 (1.36-1.76) | <0.001 | -1.53 (-2.25 to -0.82) | <0.001 | -0.99 (-1.54 to -0.45) | <0.001 |
|  | Good | Reference (1) |  |  |  | Reference (0) |  |  |  |
| Muscle strength | Weak | 1.28 (1.07-1.53) | 0.006 | 1.24 (1.08-1.42) | 0.002 | -0.71 (-1.35 to -0.07) | 0.029 | -0.92 (-1.49 to -0.34) | 0.002 |
|  | Good | Reference (1) |  |  |  | Reference (0) |  | Reference (0) |  |
| Combination of physical performance and muscle strength | Both weak and poor | 1.98 (1.52-2.57) | <0.0001 | 1.72 (1.42-2.08) | <0.0001 | -1.75 (-2.74 to -0.75) | 0.0001 | -1.71 (-2.52 to -0.90) | 0.0003 |
|  | Poor performance only | 1.45 (1.16-1.82) |  | 1.52 (1.30-1.78) |  | -1.79(-2.71 to -0.86) |  | -0.29 (-1.34 to -0.12) |  |
|  | Weak strength only | 1.17 (0.95-1.44) |  | 1.19 (0.98-1.44) |  | -0.64(-1.30 to 0.02) |  | -1.75 (-0.98 to 0.40) |  |
|  | Both good function | Reference (1) |  | Reference (1) |  | Reference (0) |  | Reference (0) |  |

- Poor physical performance: gait speed ≤0.8 m/s. Weak muscle strength: handgrip strength <27 kg for males and <16 kg for females.

**S. Table 2b:** subgroup analysis by sex showing the association between depressive symptoms and physical function

| Male | | | | | | | |
| --- | --- | --- | --- | --- | --- | --- | --- |
| Depressive symptoms (CES-D 10≥8) | | Poor physical performance | | Weak muscle strength | | Decline in physical function | |
|  |  | OR (95% CI) | p-value | OR (95% CI) | p-value | OR (95% CI) | p-value |
| Depressive symptoms | Yes | 1.53(1.29-1.80) | <0.001 | 1.11(0.97-1.27) | 0.134 | 1.79(1.31-1.96) | <0.001 |
|  | No | Reference (1) |  | Reference (1) |  | Reference (1) |  |
| Female | | | | | | | |
| Depressive symptoms (CES-D 10≥8) | | Poor physical performance | | Weak muscle strength | | Decline in physical function | |
|  | Yes | OR (95% CI) | p-value | OR (95% CI) | p-value | OR (95% CI) | p-value |
| Depressive symptoms | No | 1.45(1.28-1.63) | <0.001 | 1.11(0.99-1.25) | 0.065 | 1.65(1.54-1.80) | <0.001 |
|  |  | Reference (1) |  | Reference (1) |  | Reference (1) |  |

- Poor physical performance: gait speed ≤0.8 m/s. Weak muscle strength: handgrip strength <27 kg for males and <16 kg for females.

**S. Table 3:** Sensitivity analysis showing the association between physical performance, muscle strength, and depressive symptoms estimated from adjusted GEE model of repeated measure exposure and outcome after excluding participants with depressive symptoms at baseline.

| Physical function | | Depressive symptoms | |
| --- | --- | --- | --- |
|  |  | OR (95% CI) | p-value |
| Physical performance | Poor | 1.50 (1.33-1.68) | <0.001 |
|  | Good | Reference |  |
| Muscle strength | Weak | 1.24 (1.10-1.40) | 0.001 |
|  | Good | Reference |  |
| Combination of physical performance and muscle strength | Both weak and poor | 1.72 (1.44-2.05) | <0.0001 |
|  | Poor performance only | 1.44 (1.25-1.67) |  |
|  | Weak strength only | 1.18 (1.01-1.38) |  |
|  | Neither weak nor poor (both good physical function) | Reference (1) |  |

- Poor physical performance: gait speed ≤0.8 m/s. Weak muscle strength: handgrip strength <27 kg for males and <16 kg for females

**S. Table 4:** Sensitivity analysis showing the association between depressive symptoms and physical function as a repeated measure exposure and outcome after excluding participants with poor physical function at baseline.

| Depressive symptoms (CES-D 10≥8) | | Poor physical performance | | Weak muscle strength | | Decline in physical function* | |
| --- | --- | --- | --- | --- | --- | --- | --- |
|  |  | OR (95% CI) | p-value | OR (95% CI) | p-value | POR (95% CI) | p-value |
| Depressive symptoms | Yes | 1.55(1.38-1.73) | <0.001 | 1.15(1.03-1.29) | 0.012 | 1.69(1.58-1.81) | <0.001 |
|  | No | Reference (1) |  | Reference (1) |  | Reference (1) |  |

- POR: proportional odds ratio
- *Decline in physical function as ordinal outcome (good/normal physical function, poor physical performance or weak muscle strength and both poor performance and weak strength)

**S. Table 5:** Sensitivity analysis estimating the association between depressive symptoms and physical function, after additionally adjusting for social support (n=10,110)

| Exposure | | | Depressive symptoms | |
| --- | --- | --- | --- | --- |
|  |  |  | OR (95% CI) | p-value |
| Combination of physical performance and muscle strength | Both weak and poor | | 1.90 (1.56-2.32) | <0.0001 |
|  | Poor performance only | | 1.44 (1.22-1.69) |  |
|  | Weak strength only | | 1.20 (1.01-1.43) |  |
|  | Neither weak nor poor (both good physical function) | | Reference (1) |  |
| Depressive symptoms (CES-D 10≥8) | |  | Decline in physical function* | |
|  |  |  | POR (95% CI) | p-value |
|  |  | Yes | 1.69(1.58-1.81) | <0.0001 |
|  |  | No | Reference (1) |  |

- Poor physical performance: gait speed ≤0.8 m/s. Weak muscle strength: handgrip strength <27 kg for males and <16 kg for females
- POR: proportional odds ratio
- *Decline in physical function as ordinal outcome (good/normal physical function, poor physical performance or weak muscle strength and both poor performance and weak strength)
- Models were adjusted for age, sex, educational status, alcohol use, BMI, smoking, baseline cognition, living arrangement, racial category, multimorbidity, polypharmacy and social support.
- Social support: defined based on the Lubben Social Network Scale (LSNS-6), and a value of≥12 out of 30 points was considered good social support for the ALSOP sample (Australian participants).

**S. Table 6:** Sensitivity analysis showing the association between depressive symptoms and physical function and interaction with diet quality, physical activity and pain symptoms

| Variables | | Depressive symptoms | |
| --- | --- | --- | --- |
|  |  | OR (95% CI) | p-value |
| Physical performance and diet quality | Poor performance * pro-inflammatory | 0.86 (0.67 - 1.10) | 0.238 |
|  | Good performance * anti-inflammatory | Reference |  |
| Physical performance and physical activity | Poor performance *low physical activity | 1.06 (0.84 - 1.34) | 0.616 |
|  | Good performance * moderate physical activity | Reference |  |
| Physical performance and level of pain | Poor performance * mild pain | 2.12 (0.90 - 4.96) | 0.318 |
|  | Poor performance * moderate pain | 2.21 (0.95 - 5.16) |  |
|  | Poor performance * severe pain | 1.96 (0.79 - 4.84) |  |
|  | Good performance * no pain | Reference |  |
| Muscle strength and diet quality | Weak strength * pro-inflammatory | 1.08 (0.83 -1.40) | 0.576 |
|  | Good strength * anti-inflammatory | Reference |  |
| Muscle strength and physical activity | Weak strength *low physical activity | 0.92 (0.71 -1.18) | 0.494 |
|  | Good strength *moderate physical activity | Reference |  |
| Muscle strength and level of pain | Weak strength * mild pain | 1.00 (0.43 - 2.33) | 0.181 |
|  | Weak strength * moderate pain | 1.16 (0.50 - 2.70) |  |
|  | Weak strength * severe pain | 0.71 (0.29 - 1.74) |  |
|  | Good strength * no pain | Reference |  |
| Physical function and diet quality | Both poor performance and strength * pro-inflammatory diet | 0.87 (0.61 - 1.25) | 0.156 |
|  | Poor performance and good strength * pro-inflammatory diet | 1.06 (0.78 - 1.45) |  |
|  | Poor strength and good performance * pro-inflammatory diet | 1.34 (0.98 - 1.93) |  |
|  | Good physical function*anti-inflammatory diet | Reference |  |
| Physical function and physical activity | Both poor performance and strength * low | 0.94 (0.67 - 1.33) | 0.759 |
|  | Poor performance and good strength * low | 1.08 (0.79 -1.46) |  |
|  | Poor strength and good performance * low | 0.88 (0.63 -1.22) |  |
|  | Good physical function*moderate | Reference |  |
| Physical function and level of pain | Both poor performance and strength* mild pain | 1.17 (0.44 -3.12) | 0.234 |
|  | Both poor performance and strength* moderate pain | 1.33 (0.50 -3.51) |  |
|  | Both poor performance and strength* severe pain | 0.87 (0.30 -2.54) |  |
|  | Poor performance * mild pain | 3.34 (0.80 -14.01) |  |
|  | Poor performance * moderate pain | 4.13 (0.99 -17.16) |  |
|  | Poor performance * severe pain | 3.48 (0.79 -15.33) |  |
|  | weak strength * mild pain | 0.94 (0.35 - 2.52) |  |
|  | weak strength * moderate pain | 1.28 (0.48 -3.40) |  |
|  | weak strength * severe pain | 0.56 (0.18 -1.68) |  |
|  | Good physical function*no pain | Reference |  |

*two-way interaction, Diet quality: based on dietary inflammatory score (anti-inflammatory for negative values and pro-inflammatory for positive values). Intensity of physical activity in a typical week: low (never, rare or light) and moderate (moderate to vigorous), Pain level in the subgroup of people who experienced pain very often was categorised based on the British Society of Pain Numerical Rating Scale (NPRS-11). Poor physical performance: gait speed ≤0.8 m/s. Weak muscle strength: handgrip strength <27 kg for males and <16 kg for females.


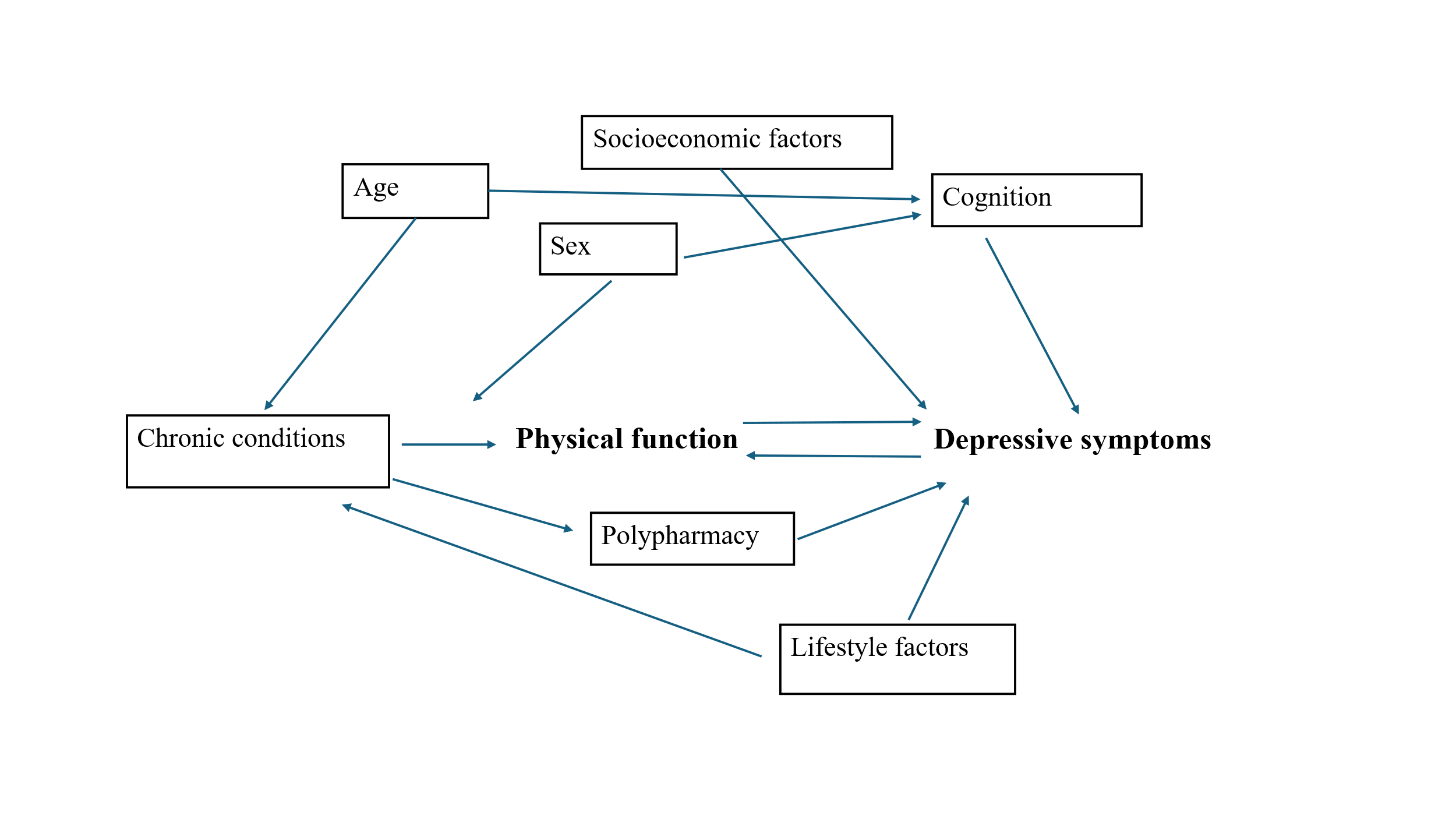
**S. Figure 1**: Factors affecting physical function and depressive symptoms in later life.


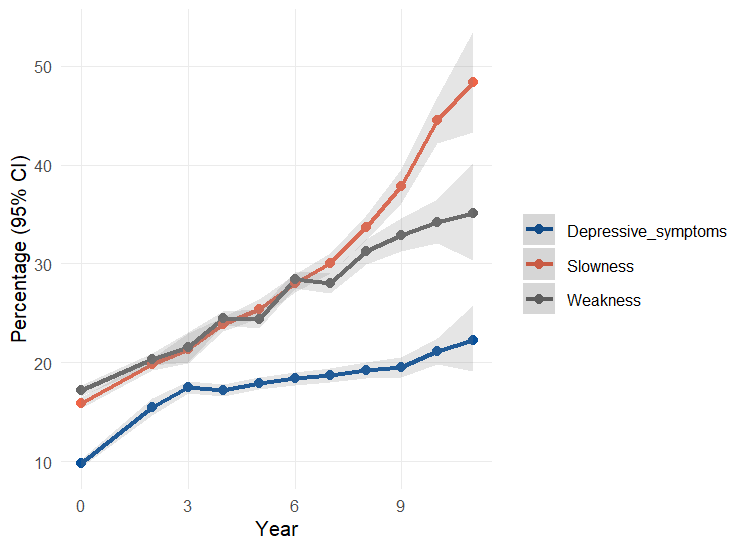


**S. Figure 2**: Proportion of depressive symptoms, slow gait speed and weak grip strength over 11 years of follow-up
